# Can Economic Analysis Contribute to Disease Elimination and Eradication? A Systematic Review

**DOI:** 10.1371/journal.pone.0130603

**Published:** 2015-06-12

**Authors:** Elisa Sicuri, David B. Evans, Fabrizio Tediosi

**Affiliations:** 1 ISGlobal, Barcelona Ctr. Int. Health Res. (CRESIB), Hospital Clínic—Universitat de Barcelona, Barcelona, Spain; 2 Department of Epidemiology and Public Health, Swiss Tropical and Public Health Institute, P.O. Box, CH-4002 Basel, Switzerland; 3 University of Basel, P.O. Box, CH-4003 Basel, Switzerland; University of Waterloo, CANADA

## Abstract

**Background:**

Infectious diseases elimination and eradication have become important areas of focus for global health and countries. Due to the substantial up-front investments required to eliminate and eradicate, and the overall shortage of resources for health, economic analysis can inform decision making on whether elimination/eradication makes economic sense and on the costs and benefits of alternative strategies. In order to draw lessons for current and future initiatives, we review the economic literature that has addressed questions related to the elimination and eradication of infectious diseases focusing on: why, how and for whom?

**Methods:**

A systematic review was performed by searching economic literature (cost-benefit, cost-effectiveness and economic impact analyses) on elimination/eradication of infectious diseases published from 1980 to 2013 from three large bibliographic databases: one general (SCOPUS), one bio-medical (MEDLINE/PUBMED) and one economic (IDEAS/REPEC).

**Results:**

A total of 690 non-duplicate papers were identified from which only 43 met the inclusion criteria. In addition, only one paper focusing on equity issues, the “for whom?” question, was found. The literature relating to “why?” is the largest, much of it focusing on how much it would cost. A more limited literature estimates the benefits in terms of impact on economic growth with mixed results. The question of how to eradicate or eliminate was informed by an economic literature highlighting that there will be opportunities for individuals and countries to free-ride and that forms of incentives and/or disincentives will be needed. This requires government involvement at country level and global coordination. While there is little doubt that eliminating infectious diseases will eventually improve equity, it will only happen if active steps to promote equity are followed on the path to elimination and eradication.

**Conclusion:**

The largest part of the literature has focused on costs and economic benefits of elimination/eradication. To a lesser extent, challenges associated with achieving elimination/eradication and ensuring equity have also been explored. Although elimination and eradication are, for some diseases, good investments compared with control, countries’ incentives to eliminate do not always align with the global good and the most efficient elimination strategies may not prioritize the poorest populations. For any infectious disease, policy-makers will need to consider realigning contrasting incentives between the individual countries and the global community and to assure that the process towards elimination/eradication considers equity.

## Introduction

The major global health achievements of the last century were possible due to improvements in health technologies and services consequent to advances in knowledge, science and technology, building on improvements in socio-economic conditions [1, 2]. The links between health improvements and socio-economic development are well established and the relationship is complex and bi-directional. For example, increasing incomes, education and other forms of social development improve living conditions and reduce risks to health. They allow people to take more responsibility for, and invest in their own health. On the other hand, improved health also allows people to earn more and improve their own living standards [3–7]. There is no doubt, however, that increased coverage of many types of health interventions—vaccines, safe childbirth delivery, treatment for tuberculosis, malaria and HIV/AIDS and prevention of cardiovascular disease to name a few—have also contributed to worldwide health improvements.

Economic analysis has facilitated a better understanding of the relationship between economic growth and health, but has also helped to make the case that investments in health produce excellent returns not just in terms of improved morbidity and mortality but also through their impact on the economic wellbeing. Economic analysis has now been extended to questions surrounding one of the most pressing global health challenges: should countries press for the elimination and eradication of infectious diseases and if so, how? Partly because of the heavy initial investments required, there are a number of areas in which economics can inform these important decisions.

We, therefore, undertook a systematic review of the literature to explore how economic analysis has to date contributed to inform the debate about the elimination/eradication of different infectious diseases. We focus particularly on whether there are common strands across diseases in the way this analysis has sought to answer three questions: why eliminate, how, and for whom? [8].

### The context: infectious diseases elimination/eradication

The proportion of the global burden of disease attributable to infectious diseases has decreased considerably in the last two decades [9]. The reasons for this are beyond the scope of this paper, but are complex and linked to improved socio-economic conditions, disease control technologies, and increased political will to reduce the burden of infectious diseases [10, 11].

Disease elimination has been defined as a reduction to zero of the incidence of infection caused by a pathogen in a defined geographical area, while eradication is a permanent reduction to zero of the global incidence [12]. Interventions are needed after elimination to prevent the reestablishment of transmission but, in principle, no longer needed after eradication.

Elimination and eradication are biologically feasible when there are safe and effective tools able to interrupt transmission; no animal or environmental *reservoirs*; adequate public health and health system infrastructures, sufficient funding and sustained political/societal will are also required [13].

The investments needed in the early years of elimination/eradication are generally much larger than those involved in running a routine control programme so economics, a science of choice in the face of limited resources, can contribute to inform decisions.

Polio eradication is close to being achieved and a plan for the eradication of measles and rubella is under development [14, 15]. Malaria is another disease where eradication has re-emerged as a global goal [16]. The vision of eliminating and eradicating selected neglected tropical diseases (NTDs) has also gathered momentum over recent years. In 2011, the WHO Strategic and Technical Advisory Group for Neglected Tropical Diseases and its partners adopted a roadmap for the elimination and eradication of 12 NTDs by 2020—rabies, blinding trachoma, endemic treponematoses, leprosy, chagas disease, human african trypanosomiasis, visceral leishmaniasis, dracunculiasis, lymphatic filariasis, onchocerciasis, schistosomiasis, and dengue [17].

## Methods

### Search strategy and selection criteria

We conducted a systematic literature review in April 2013 of articles published between 1980 and the end of March 2013 retrieved from a bio-medical (PubMed/Medline), a general (SCOPUS) and an economic (IDEAS—a Research Papers in Economics, REPEC—hosted service) database. The year in which smallpox was declared eradicated, 1980, was considered as a convenient starting year for this review because economists started to be increasingly interested in disease elimination thereafter.

Different search strategies were used in the bio-medical, general and in economic databases. Key words used in the first case were: ((eliminat* OR eradicat*) AND (cost* OR economic)). In the second case key words used were: (eliminate* OR eradicate*).

**Fig 1:**
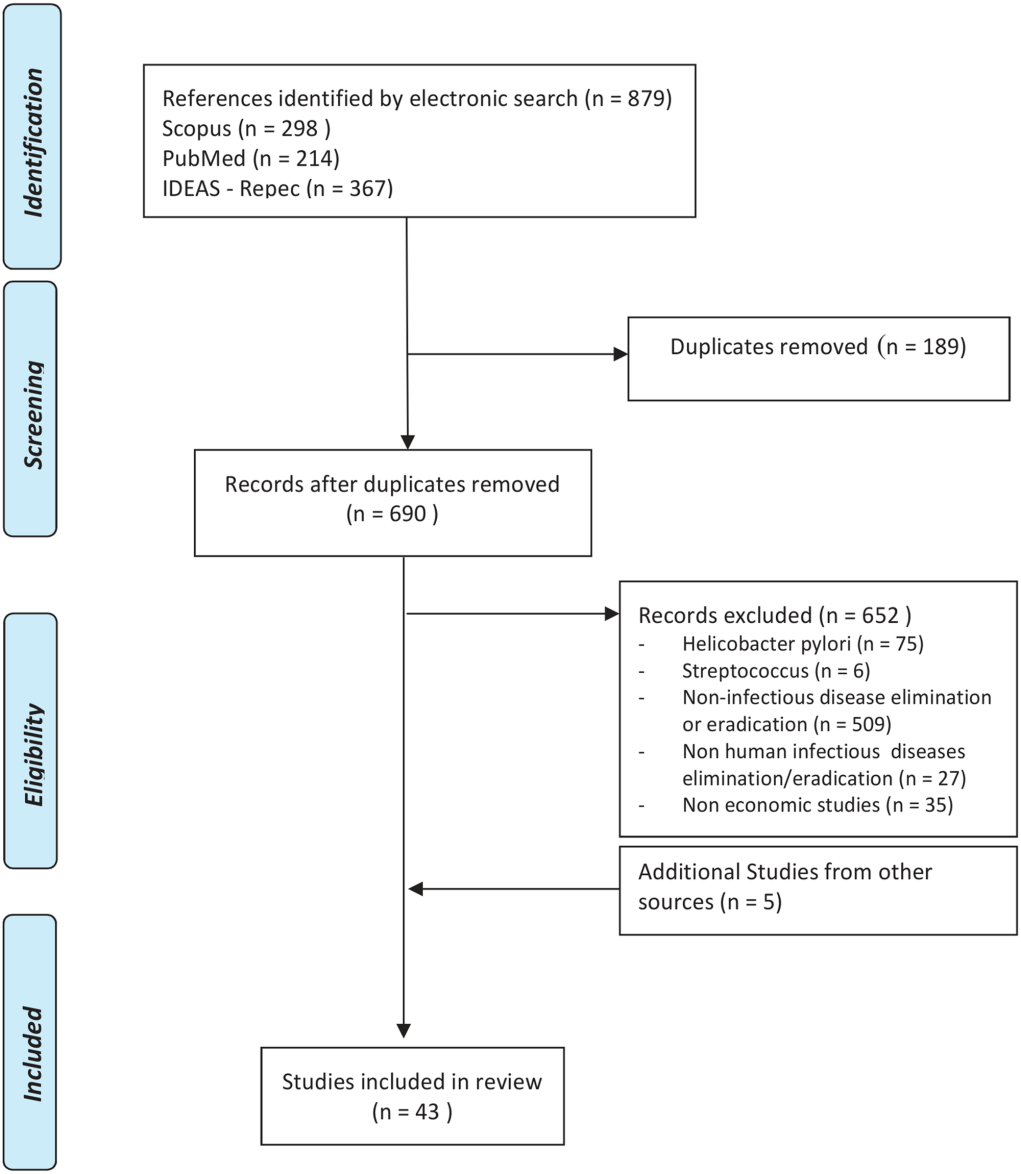
Preferred Reporting Items for Systematic reviews and Meta-Analyses (PRISMA) Diagram.

When the same research appeared both as working paper and as article, the latter was selected. Conference proceedings, comments and letters were excluded from the search. From the extracted articles only economic studies focused on the elimination/eradication of infectious diseases affecting human populations were selected (Fig 1). For equity issues a separate search was conducted with the words (equity) AND (eliminat* OR eradicat*) in all databases.

## Results

### Summary of studies

A total of 690 articles were identified after discarding 189 duplicates. Papers on Helicobacter pylori, Staphylococcus aureus and Streptococcus were discarded because they referred to elimination/eradication in individuals or small communities; so were papers on non-infectious and non-human infectious disease. That left only 38 articles, but their bibliographies revealed another 5 that had not been found in the initial search. Fig 1 summarises this search.

In the case of equity only one article, which was extracted both from SCOPUS and PubMed data bases, met the inclusion criteria. This search was not included in Fig 1.

The results of the literature review were organized by the three key questions described earlier: why, how and for whom to eliminate or eradicate? [8]. The studies dealing with the “why” question compare costs with benefits. The “how” question assesses which intervention/s or strategy/ies should be adopted using economic criteria; how to generate incentives for each country to eliminate; how many resources would be required; and how these resources could be mobilized. The “for whom” question assesses who would benefit from eradication, and the likely impact on equity and fairness.

We also classified the papers according to the type of economic issues that were considered:

- Impact of elimination/eradication on GDP growth or on its determinants—social and human capital accumulation
- Theoretical analyses using game theory
- Financial and economic costs of elimination/eradication
- Cost-benefit or cost-effectiveness analyses.

The articles aimed at assessing the impact of disease elimination/eradication on economic growth/development were analysed tabulating the following aspects:

- Main hypothesis tested/research question
- Main findings
- Type of study
- Approach used
- Methodology
- Counterfactual

The articles classified as costing studies, cost-benefit and cost-effectiveness analyses were analysed tabulating the following aspects:

- Hypothesis tested
- Main findings
- Perspective of analysis
- Costs included
- Economic benefits measured
- Health outcomes measured

Articles exploring the question “how to eliminate/eradicate” were analysed tabulating two main aspects: role of incentives and financial issues associated with elimination/eradication.

Review or opinion articles that we found during the literature search were not tabulated but we draw on them in the introduction and discussion sections of this manuscript.

### Why eliminate or eradicate?

Elimination and eradication are associated with high risks of failure in terms of disease re-emergence and require substantially higher investments than routine control activities at least initially. The health and economic benefits therefore need to be higher to justify the higher costs in the context of scarce resources and competing health problems [18]. There is considerable uncertainty about the time path, costs and outcomes of elimination and eradication strategies, so a number of studies have developed cost estimates for a number of different scenarios [19]. The various authors have also used varying methodologies in estimating costs and benefits, such as the extent to which they have discounted future benefits which accrue in perpetuity, and in the treatment of intangible costs and benefits such as anxiety or pain associated with an elimination program and the benefit of the security when a health risk no longer exists [20, 21].

To take a complete economic view of elimination/eradication would require a cost-benefit or cost-effectiveness analysis [22], but many of the studies reviewed did not undertake a complete assessment (Table 1). For example, many considered only the costs of elimination—as in the case of lymphatic filariasis either in specific countries or globally [23–25]; HIV in South Africa [26]; schistosomiasis from the Guangxi region in China [27]; visceral leishmaniasis in Bangladesh, India and Nepal [28]; and leprosy globally [29]. For malaria, Sabot et al. presented the first attempt to model costs and benefits of elimination campaigns using data from eight case studies. The probability that elimination would be cost-saving compared to control ranged from 0% to 42% [22]. This does not mean that elimination should be rejected as an option because it would bring many health benefits to the affected populations, but only that it will not be “self-financing” in the long run.

**Table 1:** Why to eliminate/eradicate: costs, benefits and economic evaluations of eliminating/eradicating infectious diseases.

Study and Year	Perspective	Content	Costs included	Health outcomes	Economic benefits	Methodological approach	Main Findings
Bart, 1996 [64]	Health system	Economic evaluation of the **poliomyelitis** eradication initiative to facilitate national and international decision-making on financial support.	Programme costs, treatment and rehabilitation costs, and vaccine costs included stratified by developing and industrialized countries	Number of cases of paralytic poliomyelitis	Treatment and rehabilitation costs	CBA. The base case examined the net costs and benefits during 1986–2040 based on differential costs for oral poliovirus vaccine and its delivery in industrialized and developing countries and ignored benefits from reductions in direct costs for treatment and rehabilitation	The "break-even" point at which benefits exceeded costs was the year 2007, with savings of US$ 13600 million by the year 2040. Results were robust to large variations in several factors affecting costs and benefits. Poliomyelitis Eradication Initiative was economically justified
Kim, 1997 [35]	Health system	Comparison between expenditure on Global **dracunculiasis** Eradication Campaign (GDEC) activities and estimates of increased agricultural production due to reductions in infection-related morbidity	The estimated expenditures for GDEC include costs incurred by foundations, NGOs, as well and by the WHO between 1987–1998	Number of cases	Estimate of productivity loss averted	CBA using a project horizon of 1987–1998. Costs based on data from Global 2000, UNICEF, and WHO. Productivity estimates based on the Cobb-Douglas production function	The Economic rate of return was 29%, under the assumption of 5 weeks average degree of incapacitation caused by Guinea worm infection. Elimination to be achieved in Sudan by the year 2001 for economic returns to be consistent with those obtained in other endemic countries
Miller, 1998 [33]	Societal	Demonstration of the potential value of **measles eradication** by identifying the potential cost-savings to the United States resulting from measles eradication	Costs of vaccine and vaccination, travel, indirect costs and costs of adverse events management	Number of cases	Costs of measles disease and outbreak control costs	CBA	Measles eradication would save $45 million annually. If achieved by the year 2010, the US would save up to $4.1 billion. Intensification of measles control efforts in the US would have minimal marginal benefits on disease burden reduction
Acharya, 2002 [32]	Health system (PAHO)	Cost-effectiveness of **measles elimination** in *Latin America* and the *Caribbean*	Costs of routine vaccination and costs of follow up campaigns (vaccine delivery, admin costs, mobilization, adverse events management)	Number of cases modelled from available information	Costs of routine vaccination	Prospective CEA of strengthening immunisation based on PAHO costs and health outcomes based on continuation of past trends	Vaccination for measles elimination costs US$ 244 incremental million and prevents a case of measles at the cost of US$ 71.75 and a death at the cost of US$ 15,000. It saves a total of US$ 208 million in treatments costs
Khan, 2003 [65]	Health system	Estimate global economic costs and benefits of **polio** vaccination and eradication by six geographic regions of the world (AFR, AMR, EMR, EUR, SEAR and WPR)	Cost of polio vaccination, eradication and treatment per case estimated using the cost per dose parameters and actual eradication costs reported by the WHO regions	Number of cases and DALYs	Polio medical care cost averted	CEA and cost savings estimates with pre-vaccination polio incidence rates in USA and Italy used to predict the cases that would have occurred in the world for the years 1970–2050 in the absence of immunization	Globally, polio program will cost US$ 67 billion if vaccination is discontinued after 2010. The medical care cost savings will be more than US$ 128 billion, with polio eradication paying for itself in the long run. The program will also prevent 855000 deaths, 4 million paralysis cases and 40 million DALYs over the years 1970–2050
Ramzi, 2005 [23]	Health system, government	Estimate costs for **lymphatic filariasis** elimination program in *Egypt* in 2000–2001	All costs, recurrent and capital, associated with 2 rounds mass drug administration	None	None	Cost data retrospectively gathered from local, regional and national Ministry of Health and Population records. The total estimates for each governorate based on data from a representative district and combined with national programme estimated data	The overall total and government costs for treatment at a coverage of 86% (year 2000) were US$ 3,181,000 and US$ 2,412,000, respectively. In 2001, with a coverage of 88%, total costs were US $3,109,000 while government costs were US $2,331,000. In 2000, the average Total and Government costs per treated subject were US $1.77 and $1.34, respectively. These costs decreased to US $1.34 and $1.00 in 2001
Thompson, 2007 [66]	Health system	Economic evaluation of worldwide eradication of wild **polioviruses** against control in the four remaining endemic areas where elimination has not been achieved yet (Afghanistan, Pakistan, India and Nigeria).	Vaccination and surveillance costs	Paralytic poliomyelitis cases	Treatment costs of cases averted	CEA, based on a dynamic model of the current endemic areas in India. An extended model assesses the economic implications and disease burden of a change in policy from eradication to control	A control routine immunisation policy for 20 years with costs of approx $3500 million may lead to 200 000 paralytic poliomyelitis cases every year; a control policy keeping the number of cases at about 1500 per year could cost around $10 000 million. Immunization intensity must be increased to achieve eradication implying to pay higher short-run costs than currently spent
Michael, 2008 [67]	Ministry of Health	Different scenarios of global disease control and elimination/eradication of **Lymphatic filariasis** are compared.	Unit costs taken from published sources (excluding cost of drugs donated) and total costs based on modelling	Number of individuals cured of microfilarial infection (estimated through mathematical modelling)	Not included	CEA	In 10 years control more cost-effective than elimination because of high marginal cost of curing the last few individuals to achieve elimination and low additional health outcomes
Chu, 2010 [24]	Individuals and health system	Economic benefits, in terms of treatment savings due to the first 8 years of Global Programme to Eliminate **Lymphatic Filariasis** (GPELF) (2000–2007) through annual mass drug administration (MDA)	Costs of MDA not estimated	Individuals and person-years protected from acquiring infection or from disease progression	Individual direct and indirect and health system savings from infection or disease progression averted	The number of cases averted quantified by assuming a 10% infection rate and by considering that just a fraction seeks treatment at public health centres. Associated savings in terms of treatment costs refer to medicines, consultation fees, transport, food, accommodation and indirect costs of lost-labour	US$21.8 billion of direct economic benefits gained over the lifetime of 31.4 million individuals treated. Of these, over US$2.3 billion is a consequence of nearly 3 million babies born in areas free of LF. US$19.5 billion is the lifetime economic benefit from stopping disease progression of more than 28 million infected individuals. US$2.2 billion are health system savings due to reduced LF morbidity
Sabot, 2010 [22]	Health system/government	To present a conceptual framework to analyse short to medium term financial savings consequent to **malaria** elimination based on data from eight countries either pursuing or recently achieving elimination	Costs data from several sources, including both actual and programmatic costs either from budgets or mathematical models	Incremental reduction in malaria incidence comparing elimination and control scenarios	Benefits estimated based on yearly cost data and assigned to one of the three elimination phases: baseline, interruption of transmission (10 years) and post-elimination (15 years)	Review of published works and datasets from elimination programmes in eight countries. Costs modelled to the elimination phases context. Minimum-maximum sensitivity analysis was applied	The probability that elimination is cost-saving over 50 years ranged from 0% to 42%. Financial savings should not be a primary rationale for elimination, but elimination still is a worthy investment if total benefits are sufficient to outweigh marginal costs
Adhikari, 2010 [36]	Societal	A cost benefit analysis of **elimination of kala-azar (KA)** in *Nepal*	Costs of interventions are the values of inputs used to control KA for a year, including costs of treatment and of prevention	Number of cases prevented due to interventions of KA in a year	Estimates of productivity gains due to cases prevented and treatment costs saved.	CBA	Total discounted net benefit of KA intervention was in Nepalese Rupees (NRs) 65,287 million with 35% investment return. Every rupee invested in KA intervention at present will yield NRs 71. Elimination of KA is a good investment opportunity
Duintjer Tebbens, 2010 [68]	Health system	Economic analysis of the Global **Polio Eradication** Initiative (GPEI) and full consideration of post-eradication policies. GPEI consists in a global scaling up of polio vaccination	Vaccine and non-vaccine costs child, including for personnel, training, transportationand cold chain, building andequipment	Disability-adjusted life-years associated paralyticpoliomyelitis cases	Average direct treatment costs associated with one paralytic poliomyelitis case	CEA, CBA. GPEI compared against routine vaccination. Costs of eradication were based on actual and projected expenditures Polio incidence estimated using a dynamic infection transmission model and costs based on numbers of vaccinated children	GPEI vs routine vaccination was highly cost-effective. Incremental net benefits of the GPEI between 1988 and 2035 were of 40–50 billion dollars (2008 US dollars). Despite the high costs of achieving eradication in low-income countries, these account for 85% of the total net benefits generated by the GPEI
Levine, 2011 [30]	Societal	Economic evaluation of **measles eradication** with costs of vaccination collected in Bangladesh, Brazil, Colombia, Ethiopia, Tajikistan, and Uganda, comparing eradication with 3 scenarios: (1) 90% mortality reduction by 2013 (baseline);(2) 95% mortality reduction by 2015;(3) 98% mortality reduction by 2020. Data globally extrapolated	Annual program costs were summed for the immunizationuntil measles eradication. Costs of routine immunization estimated through ingredient approach. Indirect and societal costs estimated based on interviews and/or published data	Measles cases, deaths avertedand DALYs averted for each strategy for each country obtained from dynamic transmission modelling	The direct costs and/or savings from not treating averted measlescases were estimated using available data on the costs oftreatment	Prospective CEA comparing eradication with different scenarios of mortality reduction at different time points and health outcomes based on a transmission model. A global analysis was undertaken by using the existing transmission model	Measles eradication by 2020 was found to be the most cost-effective scenario, both in the six countries and globally. Eradicating measles by 2020 is projected to cost an additional discounted $7.8 billion andavert a discounted 346 million DALYs between 2010 and 2050
Babigumira, 2011 [31]	Health system and household	Economic evaluation of **measles elimination** in *Uganda* as part of a global eradication program based on immunization scale-up by comparing different scenarios: 90% mortality reduction by 2013; 95% mortality reduction by 2015; 98% mortality reduction by 2020; elimination in 2020; and elimination in 2025.	The cost of each scenario was estimated as the sum of: (1) cost of measles immunization activities, including measles surveillance and outbreak response;and (2) household costs of immunization	Measles incidence, cases of measles averted, deaths averted, DALYs averted (estimated through modelling)	The cost savings fromreduced measles treatment for cases averted were calculated by multiplying the average cost of treatment	Prospective CEA based on a transmission model and on immunisation costs associated with progressive scale-up	Measles elimination by 2020 was the most cost-effective scenario compared to different scenarios of mortality rate reduction and of later elimination.
Bishai, 2012 [34]	Societal	To estimate the global cost-effectiveness of **measles** eradication versus control	Total costs include costs of scaling up routine vaccination, other immunization activities, outbreak control, routine surveillance, health sector costs of treating measles cases, and societal costs of lost productivity	Measles DALYs	Costs for measles control saved	CEA based on a dynamic age-tiered measles transmission model for 6 countries (Bangladesh,Brazil, Colombia, Ethiopia, Tajikistan, and Uganda) extrapolated globally. Alternatives compared were constant vaccine coverage at 2010 levels, eradication by 2020, eradication by 2025, 95% mortality reduction by 2015, and 98% mortality reduction by 2020. Cumulative discounted societal costs, caseloads, lives, and DALYS saved with each policy option were compared	Strategies to eradicate measles in Bangladesh, Ethiopia, and Uganda cost more than twice as much as control strategies, but have similar costs per death averted. For Brazil, Colombia and Tajikistan, eradication by 2020 would prevent deaths and save $800 million more than measles control from 2010–2050. Measles eradication and measles control are both cost effective and have equivalent costs per life saved in low income countries, but high income countries derive savings only if measles is eradicated and imported cases stop
Goldman, 2007 [25]	National Lymphatic Filariasis programme	Estimate of annual mass drug administration (MDA) costs for the **elimination of lymphatic filariasis** (LF) in 7 countries	Financial and economic (including value of donated goods, such as drugs) costs considered. Recurrent costs included and capital costs	Number of persons treated	Not quantified	Ingredient approach used for cost estimates per activity carried out. Capital costs annualized. Costs estimated per MDA round	Financial costs per person treated ranged $ 0.06–2.23; economic costs ranged $ 0.40–5.87. MDA for LF elimination appeared inexpensive compared to most other public health programs
Wagner, 2010 [26]	Health system	Estimate of treatment costs associated with **elimination of HIV in South Africa** referred to a previous study (Granich et al [69]	Maximum cost of treating a patient is US$1163 and $4083 per year with first (97% of patients) and second-line drugs (3% of patients), respectively	Number of individuals on antiretroviral treatment	Not quantified	Cost curve constructed for 40 year time period based on the projected number of individuals on antiretroviral treatment	Annual costs present a pick at the year 5 but then drop considerably. The critic to Granich et al. is that costs for HIV elimination have been underestimated as they include treatment costs only ignoring, among others, screening costs
Sleigh, 1998 [27]	Health government administration	Costs of the Guangxi (China) **schistosomiasis** Control Programme for 40 years (1952–1992)	Fixed costs were excluded. Running costs were included except wages and fringe benefits for staff at provincial and prefectural levels	Not included	Not included	The total mean annual cost per county was calculated as the sum of 7 annual cost categories averaged across selected historical intervals. For grand totals the annual sums per county was multiplied by the number of counties each year.	In US$ 1991, the highest costs occurred during the period 1976–80, with a mean annual total of US$ 46,702 per county, with environmental change being the most expensive cost item. Costs declined by 33% over the last 10 years. The total cost for the whole Guangxi programme was US$ 241,944
Naik, 1998 [29]	Health system	Review of studies to explore the socio-economic factors associated with the worldwide eradication of **leprosy**		Not included	Not included	Drug and treatment costs; operational costs such as transportation, staff training and salary, laboratory and referral services	The expected cost of eliminated the disease worldwide is US$ 200 million. If operational costs included (staff training, salaries, transportation, lab and referral services) such costs may be twice or three times higher
Huda, 2012 [28]	Health system	Estimate costs associated with active case detection in national **visceral leishmaniasis (VL) elimination** programs inBangladesh, India, and Nepal	Costing ingredients included training, development and production of training materials, diagnostic kits, per diems, transport, communication and material	New VL case detected	Not included	Ingredients costing method	The cost (training costs excluded) of detecting a new VL case was of US$ 22 in Bangladesh, US$ 199 in Nepal and US$ 320 in India. Despite the cost, adequate resources for training, planning, etc were indicated as priorities
Wutzler, 2002 [37]	Societal and payer perspectives	Based on an age-structured decision analytic model, to estimate costs, benefits and cost-effectiveness of varicella immunization programme for a period of 30 years, within the framework of determining feasibility of **varicella elimination** through universal coverage	Resources used and unit costs (direct costs) for treatment and intervention of each model outcome plus the value of days off work	Varicella cases, death avoided and life year gained	Costs averted	Human capital approach for estimate of productivity loss; ingredient approach for estimate of treatment and intervention direct costs, health impact based on an age-based analytical disease model	With a routine varicella programme targeting children with a coverage level of 85%, the disease could be eliminated in 18 years. Average yearly discounted net cost savings are 51 million euros with a benefit-cost ratio of 4.12. After initial higher costs, net positive savings, both from societal and payer perspectives, can be achieved after 3 years of the programme starting

Abbreviations: CBA = cost-benefit analysis; DALY = disability-adjusted life year; CEA = cost-effectiveness analysis; MDA = mass drug administration; GPEI = global polio eradication initiative; KA = kala-azar; GPELF = global programme to eliminate lymphatic filariasis; VL = visceral leishmaniasis.

Several economic evaluations of global measles eradication or elimination indicate that the strategies are either cost-effective or cost-beneficial compared to routine immunization and mortality reduction campaigns [30–33]. These studies found that the benefit consequent to measles elimination or eradication applied to high and middle income (particularly Latin American) countries as well as to low income countries. For the former, the benefit consisted in not having to manage responses to imported measles cases; for the latter the benefit consisted in ending the need to conduct resource-intensive mortality reduction campaigns after eradication. Bishai et al. disputed the idea that measles eradication and elimination were cost-effective for low- and middle-income countries if they acted by themselves. An important reason why the global benefits of elimination and eradication outweigh the costs is because they prevent the high costs currently associated with identifying and treating imported cases of measles in high-income countries. An economic analysis from the perspective of the lower income countries themselves is less clear cut [34]. The implication is that globally it would make economic sense to eradicate measles, but low and middle income countries may have limited incentive to do so by themselves because an important part of the returns to their investments would accrue to high income countries.

The eradication of dracunculiasis has been estimated to have a high return on investment (29%) [35], and elimination of visceral leishmaniasis in Nepal was estimated to have an even higher return (35%) [36]. The benefit-cost ratio of the elimination of varicella in Germany was estimated to be 4.12 (estimates above 1 imply the investment is worthwhile on economic grounds) [37]. Elimination and eradication of lymphatic filariasis (LF) was found to be less cost-effective than control in the short term [33] although the lifetime economic benefits of reduced treatment in the first eight years of a Global Programme to Eliminate LF were estimated to be US$ 21.8 billion [24].

The studies reported here were based on models of what would happen if a disease was eliminated or eradicated. Few diseases have been eliminated or eradicated in practice, so we could find only one economic study, dated 1986, of actual elimination (of malaria from Sardinia). The same study also examined the past attempts to eliminate malaria in Sri-Lanka [38].

As we stated earlier, the reductions in treatment costs that would result from elimination or eradication are only one part of the potential economic benefits. Few studies tried to estimate the impact of elimination or eradication on economic growth through the effects on human capital accumulation (Table 2). In most of these studies elimination programmes are retrospectively used as quasi-natural experiments with the aim of identifying the causal relationship between health improvements and subsequent economic growth [39–44]. Where studies looked at the impact of past malaria or hookworm elimination campaigns on the incomes of individuals or households as opposed to economies as a whole, the results are conflicting, sometimes suggesting higher earnings and literacy rates or years of schooling in adults born after malaria elimination campaigns and sometimes results show no significant effects.

**Table 2:** Why to eliminate/eradicate: The impact of elimination/eradication on economic development, human capital accumulation.

Study and Year	Hypothesis tested/Research question Content	Main Findings	Counterfactual	Approach	Methodology used	Positive Economic impact identified?
Brown, 1986 [38]	Relationship between **malaria** eradication, population growth and economic development, represented by crop production, in *Sardinia* and *Sri-Lanka*	Effect of malaria eradication on population growth but not on economic development	Outcomes in Non-malaria areas in the two islands	Macro looking at aggregate trends	Before and after study	No
Bleakley, 2007 [43]	Economic impact of successful **eradication** of **hookworm** disease in the *American South*	Areas with higher levels of hookworm infection experienced greater increase in school enrolment, attendance and literacy after intervention, even by controlling for relevant factors. Affected cohorts showed income gains that coincided with exposure to hookworm eradication in the long term. Eradication increased return to schooling	Differential outcomes of cohort of low-infection areas	Micro, individual level combined to construct panel of average outcomes by cohort	Quasi-experimental econometric identification strategy	Yes
Bleakley, 2010 [42]	Impact of **malaria**-eradication campaigns in the *USA* (1920) and in *Brazil*, *Colombia and Mexico* (1955) on labour productivity and income of children exposed to **malaria** during adulthood	Relative to those of malaria free areas, cohorts born after eradication campaigns had higher income as adults than preceding generation. These changes coincided with childhood exposure to the campaigns rather than to pre-existing trends	Differential outcomes of cohorts born in malaria free areas	Micro, individual level	Quasi-experimental econometric identification strategy	Yes
Lucas, 2010 [44]	Impact of **malaria** eradication campaigns in *Paraguay* and *Sri-Lanka* on lifetime female education attainment	Regions with the highest pre-eradication malaria rates experienced the largest gains in education (years of completed schooling or literacy). Reducing malaria incidence by 10% leads to an increase in completed schooling of 0.1 years and an increase in the probability of being literate by 1%	Differential outcomes of Low-infection areas	Micro, individual level	Quasi-experimental; identification strategy Difference-in-difference analysis	Yes
Cutler, 2010 [41]	Analysis of the effects of childhood exposure to **malaria** national eradication campaign in *India* in the 50s on human capital accumulation and income in adulthood.	The program led to either modest increase in income for prime age men or to no improvement for women suggesting that observed effects were likely driven by increased labour market productivity. No evidence was found of increased educational attainment for men, and mixed evidence for women	Differential outcomes of Low-infection areas	Micro, individual level	Quasi-experimental econometric identification strategy; Difference-in-difference analysis	Yes, although limited
Lucas, 2011 [39]	Impact of **malaria** elimination campaign on fertility in *Sri-Lanka* in the 40s	Malaria elimination increased fertility due to both improved child survival and to a change in preferences. Fertility increases can cause a reduction in Gross Domestic Product (GDP) per capita as the size of the non-productive segment of the population increases. Malaria eradication increased female educational attainment and the net effect on GDP per capita of education and fertility effect should be positive, but are not visible in the short term	Low-infection areas	Micro, individual level	Quasi-experimental identification strategy; Difference-in-difference analysis	Yes, although delayed
Barofsky, 2011 [40]	This study evaluates the economic consequences of a **malaria** eradication campaign (1959–60) in the South Western *Ugandan district of Kigezi*	Eradication produced differential improvements in Kigezi compare to the rest of Uganda in years of schooling, literacy, and primary school completion. In addition, eradication increased income levels	Differential outcomes before and after the Campaign of areas in Uganda where the campaign was not carried out	Micro, individual level	Quasi-experimental identification strategy; Difference-in-difference	Yes
Hulden, 2012 [70]	Analysis of the association between malaria elimination and household size for 188 countries	When the average household size drops below 4 persons the probability of malaria eradication jumps dramatically and its incidence in the population drops significantly		Micro- Country level	Instrumental variables approach	Yes
Modrek, 2012 [45]	This study examines the empirical relationship between the demand for travel and malaria cases in two countries (Mauritius and Dominican Republic) around the time in which **malaria**-*elimination* campaigns were carried out	The relationships between tourist arrivals and malaria cases were statistically insignificant once confounders were accounted for, suggesting that any economic benefits from tourism derived from elimination programs are likely to be small when measured at a national level	No counterfactual	Micro	Time series methods to explain the logarithm of number of tourists in time as a function of the logarithm of number of malaria cases	Yes, but limited

Some studies suggest a possible dilemma resulting from eradication/elimination. If life expectancy increases, GDP per capita could fall unless the increase in GDP associated with a larger labour force was sufficient to offset the increase in population [39]. However, focusing on the impact of the size of the labour force on GDP through the effect of elimination/eradication on life expectancy may not always be appropriate [6]. Some health improvements may not lead to a longer life but may improve quality of life and hence the productivity of people who work. Hookworm infection is a case in point. Eliminating hookworm disease has been shown to make children learn quicker in school and increase their incomes later in life [43]. However, it does not increase life expectancy. Nevertheless, improving health without affecting life expectancy may still provide a large economic pay-off and a focus on life expectancy may miss this. When studying the impact of a malaria eradication campaign in Colombia, Bleakey noted that the elimination of *Plasmodium vivax* malaria led to more significant gains in human capital and income than the elimination of *Plasmodium falciparum*, even though *falciparum* causes more deaths than *vivax* [42].

The effects of malaria elimination campaigns on selected non-health drivers of economic growth have also been studied. The effect on tourism was estimated for Mauritius and the Dominican Republic. At the national level, and after accounting for several possible confounding factors, the impact was small and not significant [45]. From a macro-economic point of view it has been argued that there was little impact of malaria elimination on GDP growth and that other factors, such as technological change and human capital accumulation, were the major drivers of the escape from poverty in the South of Europe in the fifties [46].

### How to eliminate and eradicate?

Regional elimination and global eradication imply collective decision making, where several stakeholders have to decide on issues of collective interest in a context of different preferences and endowments. As part of the broad economic analysis, social choice theory is the study of collective decision processes and outcomes and game theory studies the strategic interdependence of individual choices and the design of collective choice rules to implement socially optimal decisions [47]. These approaches could be used to assess the success probabilities, or the impact, of different elimination or eradication strategies, to better inform a global or regional decision—e.g. should the strategy start by focusing on areas of the most intense transmission before moving to other areas? Our search did not reveal any examples of this type of analysis. However, there has been some theoretical work focused on how to encourage all of the affected countries to move towards eradication or elimination (Table 3) [19, 48–51].

**Table 3:** How to eliminate/eradicate: the role of incentives and financial issues.

Study and year	Type of study and content	Incentives	Financial issues
Barrett, 2004 [48]	Policy paper/review	As benefit can be much higher than costs, the incentives for countries to participate in an eradication initiative can be strong. Based on the smallpox experience, poor countries that may trying to eliminate might fail due to lack of expertise. Thus, eradication may require international cooperation on top of coordination. In some cases, the incentive to eliminate may depend on each country being assured that all other countries would eliminate. For this, eradication may require third party enforcement.	If poor countries are either unable or they lack the incentive to eliminate a disease on their own or as part of a coordinated effort, rich countries might have an incentive to finance the global eradication program. This way, rich countries would earn a return on their investment, making poor countries also better off.
Barrett, 2006 [49]	Policy paper on smallpox eradication	According to costs and benefits of eradication, the rich countries had an incentive to eliminate smallpox unilaterally. Many poor countries, such as India, also had an incentive to eliminate unilaterally, but lacked the capability to do so. International assistance was needed.	The weakest link nature of eradication, and the very high benefit-cost ratio (over 400:1), translate into developed country financing of elimination in developing countries. This could be achieved by coordination.
Barrett, 2007 [50]	Theoretical paper where a general epidemiology model is linked with the optimization model (constraint maximization of a socially efficient vaccination program)	A full global cost—benefit analysis is needed to determine whether eradication is a good deal overall. Being eradication an extreme goal, the analysis of an eradication program needs to begin from the perspective of where the program will end, thus from the “last mile”.	NA
Barrett, 2011 [51]	Theoretical paper on polio, modelling the cessation of vaccination after eradication linking a epidemiology, a risk of re-emergence and an economic model	The incentives to eradicate polio are closely linked to the post-eradication game. Equilibrium strategies and efficient outcomes are defined for different level of incentives and under different conditions	NA
Barham et al, 2009 [53]	Impact evaluation of conditional cash transfer program in Nicaragua to rise vaccination coverage towards a level that would be required for eradication (95% for measles, for instance)	The study finds that effects are particularly large for the children who are hard-to-reach with traditional supply-side interventions.	NA
Chesson et al, 2008 [55]	The paper evaluates the impact of greater amounts of state-level funding for syphilis elimination on syphilis rates in subsequent years in the US	NA	Higher level of funding, specifically, federally-funded syphilis elimination activities have a notable impact on syphilis rates
Geoffard et al, 1997 [54]	Theoretical paper analysing the contribution towards disease eradication of public and private interventions to increase vaccination coverage	From the public perspective, both price subsidies and mandatory vaccination programs have limited ability to achieve eradication because the increase in demand of individuals covered by the programs lowers the incentive to vaccinate for those outside the program. From the private perspective, price subsidies may make it potentially profitable for a monopolist to eradicate the disease. However, a vaccine monopolist faces a nonstandard dynamic incentive to increase markups, limiting the demand for the monopolist's product.	A deficit-financed eradication program, which spends beyond tax revenues but recoups the deficit in future generations, may improve welfare. In fact, such a program would allow for the intergenerational transfers that are necessary to pay current generations to over-vaccinate for the benefit of future generations, which although benefit, do not compensate the vaccine manufacturers.

These studies recognize that most infectious diseases are cross-border issues and that once a disease is eradicated, all countries benefit independently from their individual contributions (the public good concept). However, in order to achieve eradication (or regional elimination) all affected countries need to take actions. For countries that share borders the success of elimination depends also on the decision of the neighbors to eliminate.

Starting from a rationality assumption where stakeholders take decisions aimed at maximizing their net benefits, there are four possible outcomes:

- The net benefits of eradication for the whole world or for the countries involved in regional elimination may be negative, making control the chosen option for everyone;
- The net benefits of elimination may be so high that every country chooses to eliminate a disease unilaterally, making eradication or regional elimination the universal choice;
- It may not be worthwhile for any country to eliminate the disease unilaterally given that others have not eliminated the disease. It may also not be worthwhile for any country to eliminate the disease once all others have done so. In this case, eradication or regional elimination is a “coordination” game. There are two possible outcomes: in one, no country eliminates; in the other, every country eliminates the disease and eradication is achieved. To tip the balance towards eradication, each country would need to be convinced that all other countries will eliminate and not try to free ride on the efforts of others;
- It may not be worthwhile for the last country to eliminate a disease after all others have done so, and yet the net benefits of eradication may be positive to the world as a whole. In this case, incentives or disincentives aimed at the recalcitrant country would need to be developed.

Given that options c) and d) are the most likely, researchers are exploring if forms of global governance or international regulation within the field of international law might be appropriate [52]. The other option is that the richer countries will need to pay for the elimination efforts in poorer countries or in countries that do not choose to do it voluntarily, as happened for smallpox eradication [49].

Within country borders, the elimination of an infectious disease implies increasing the demand for preventative measures, such as vaccines. In this case, incentives are needed to raise immunization coverage that allows blocking transmission of the infection. Bahram et al. evaluated a randomized experiment showing that cash incentives paid to mothers increased vaccination coverage to 95% in rural Nicaragua [53]. Theoretical work by Geoffard and Philipson analyzed the difficulties of achieving disease eradication through vaccination using various types of incentives, highlighting that increased coverage levels reduce the incentive of those not yet vaccinated because they are less at risk of contracting the disease [54]. Chesson et al. found that greater amounts of state-level funding for syphilis elimination had a notable impact in lowering syphilis rates in subsequent years within the context of a national elimination plan in the US [55].

### For whom to eradicate?

In principle, elimination and eradication should resolve the equity/efficiency trade-off inherent in the scale-up of health interventions, in which efficiency implies quickly providing access to the easy-to-reach groups (e.g. more affluent groups in urban areas) and equity implies specifically targeting resources to poor and vulnerable groups who might be harder and more costly to reach [56]. All infectious diseases that are targeted for elimination are concentrated among the poor, so elimination/eradication would improve the health of the poor disproportionally. Disease elimination thus, interpreted through the lens of the social-choice theory described earlier, can be seen as the realization of an egalitarian policy: policies should aim either at compensating for unequal endowments for which the affected individuals are not responsible or at equalising certain “capabilities” essential for the preservation of life and the ability to freely choose one’s own way of life [57, 58]. In this respect, health is both an endowment for which individuals are at birth not responsible and a crucial constituent of human capabilities.

The literature applying these concepts to disease elimination and eradication is, however, limited apart from concerns that the process leading to disease elimination may lead to greater inequity in the short term. This may occur if countries choose to begin expensive elimination programs in less challenging areas which are usually low transmission areas where people are more affluent and healthier. The implications for equity may be even higher if elimination fails so control ends up being more effective in the areas that were relatively affluent initially, a concern that has been expressed for example with malaria elimination programmes [59]. These concerns seem to be supported by apparent small improvements in equity that have been observed with the scale up of coverage of certain malaria interventions to date, suggesting that the least affluent and most needy have proved more difficult to cover [60, 61].

## Discussion

We have examined the contribution that economic analysis has made to the question of the elimination/eradication of infectious diseases. Elimination/eradication is particularly interesting because it will require substantial initial investments, more than running routine control programs. It is also linked to the concept of public good; it will not happen unless all affected countries take a conscious decision to do it, while at the same time countries have an incentive to let others pay.

The economic literature relating to “why eliminate/eradicate” is the largest, much of it suggesting that the eventual financial returns could be high—in the long run there might be substantial savings from not having to treat people or run routine control programs. In some cases, however, this does not look likely but then there are other economic benefits in terms of increased economic productivity. A problem in this case is that these additional benefits do not necessarily accrue to the people who pay for the elimination efforts in the first place, which make financing elimination/eradication challenging.

Despite the belief of some public health experts that the economic benefits of investing in health should not be relevant to public decision-making, which should focus only on human rights to the highest attainable level of health, we suggest that national governments, global health policy makers, and donors always need to know how much a proposed course of action will cost, and what the benefits will be in terms of health and economic wellbeing. We acknowledge the importance of human rights, but it does not help to ignore the fact that resources are always limited.

Economics has contributed to addressing the question of how to invest in eradication or elimination by highlighting that there will be incentives for individuals and for countries to free-ride and that forms of incentives and/or disincentives for this behaviour will need to be developed. This requires government involvement at country level and global coordination and cooperation. It will not happen if left to the market or to individual endemic countries to act alone. While equity should be a key factor to guide the implementation of any health intervention, we found only one article analysing the impact on equity of disease elimination campaigns. However, when complemented with other evidence on the trade-offs between equity and efficiency in healthcare delivery more generally, this was sufficient to trigger a series of reflections. While there is little doubt that eliminating infectious diseases will eventually improve equity, it is not clear that equity will be improved on the path to elimination and/or eradication in the short run. Countries will have an incentive to begin with easier-to-reach areas which will often be those with higher incomes and levels of health, so specific equity strategies would need to be applied early. An interesting twist to the equity story is currently being provided by measles where the refusal to have children vaccinated among affluent people in the richest countries is proving a stumbling block to the final efforts towards eradication. The strategies adopted on the path to elimination are, therefore, particularly important and must be based not simply on considerations of cost and cost-effectiveness.

Though this was not the focus of our review, disease eradication/elimination initiatives clearly need to take into account the broad health system impacts as well which are often difficult to incorporate into an economic analysis [62, 63]. This is an added dimension that policy-makers always consider in addition to cost and impacts, and equity.

We found few articles on this aspect, and few eligible articles for this review of the economic literature in general. No article integrated the different components of costs, benefits, public good and equity issues. We found, instead, that articles published in public health journals were more focused on costs and benefits in terms of reduced treatment costs (direct benefits) while articles published in economic journals concentrated on the impact on household or national incomes (indirect benefits). None of these strands of literature included equity issues. Despite this, the literature reviewed provides some evidence of the costs of various elimination/eradication strategies, the possible impact in terms of treatment costs and sometimes incomes, and allowed us to draw some important policy conclusions. The most important one involves the need to understand and realign contrasting incentives between the motivation of individual countries and the global community if elimination/eradication is to happen. It is also critical to consider equity on the path to elimination/eradication rather than just at the end.

## Supporting Information

S1 Table PRISMA 2009 check-list. (DOCX)
